# Association of Excessive Sleepiness, Pathological Fatigue, Depression, and Anxiety With Different Severity Levels of Obstructive Sleep Apnea

**DOI:** 10.3389/fpsyg.2022.839408

**Published:** 2022-03-31

**Authors:** Karin Elisabeth Sundt Mjelle, Sverre Lehmann, Ingvild West Saxvig, Shashi Gulati, Bjørn Bjorvatn

**Affiliations:** ^1^Department of Otorhinolaryngology and Head and Neck Surgery, Haukeland University Hospital, Bergen, Norway; ^2^Department of Clinical Science, University of Bergen, Bergen, Norway; ^3^Norwegian Competence Center for Sleep Disorders, Haukeland University Hospital, Bergen, Norway; ^4^Department of Thoracic Medicine, Clinical Center for Sleep Medicine, Haukeland University Hospital, Bergen, Norway; ^5^Department of Neurology, Haukeland University Hospital, Bergen, Norway; ^6^Department of Global Public Health and Primary Care, University of Bergen, Bergen, Norway

**Keywords:** anxiety, depression, fatigue, obstructive sleep apnea, polygraphy, sleepiness

## Abstract

**Objective:**

The aim of this study was to investigate possible associations between obstructive sleep apnea and fatigue. This naturally led to considering the association between OSA and excessive sleepiness, depression, and anxiety.

**Background:**

OSA is a highly prevalent sleep disorder, associated with a risk of hypertension, cardiovascular events, daytime sleepiness, poor cognitive function, and sudden death during sleep. Both excessive sleepiness, fatigue, and symptoms of depression are frequently reported.

**Method:**

5,464 patients referred to a university hospital for obstructive sleep apnea underwent standard respiratory polygraphy. The severity of OSA was defined as either mild, moderate, or severe, using the apnea–hypopnea index. Validated questionnaires were used to assess excessive sleepiness, pathological fatigue, depression, and anxiety.

**Results:**

Nearly 70% of the patients were men, and median age was 50 (range: 16*–*89) years. One in three had moderate-to-severe OSA (AHI ≥ 15). Excessive sleepiness and pathological fatigue were present in 43 and 39%, respectively. The prevalence of possible depression and anxiety was 19 and 28%, respectively. The proportion of patients with male sex, obesity, and excessive sleepiness increased with the severity of OSA. In contrast, the proportion of patients with pathological fatigue did not increase with OSA severity, and there was a decrease in depression and anxiety with increasing OSA severity.

**Conclusion:**

Our study reports that pathological fatigue, as well as anxiety and depression, did not increase with OSA severity, whereas excessive sleepiness did. Knowledge concerning conditions related to OSA severity may be indicative of pretest probability of OSA and thus change the priority for polygraphy. Furthermore, the high prevalence rates of fatigue, anxiety, and depression among these patients warrant further investigations.

## Introduction

Obstructive sleep apnea (OSA) is a sleep disorder where airways are frequently obstructed during sleep—usually by collapsing upper airways (Veasey and Rosen, 2019). OSA is common, but the prevalence varies between different studies. A large Swiss population-based study among subjects aged 40–85 years found that 49% of men and 23% of women have moderate-to-severe (apnea–hypopnea index, AHI ≥ 15) OSA (Heinzer et al., 2015). A Norwegian population-based study among subjects aged 30–65 years reported a prevalence of 8% for moderate-to-severe OSA and 16% for mild-to-severe OSA (AHI ≥ 5; Hrubos-Strom et al., 2011). OSA is associated with an increased risk of hypertension, cardiovascular events (Peker et al., 2002), daytime sleepiness (Bjorvatn et al., 2015), poor cognitive function (Naegele et al., 1995), and sudden death during sleep (Gami et al., 2005). Among well-known risk factors are obesity (Young et al., 2004; Tuomilehto et al., 2013; Heinzer et al., 2015), male sex (Young et al., 2004; Sforza et al., 2011; Heinzer et al., 2015), and increasing age (Young et al., 2004; Edwards et al., 2010; Heinzer et al., 2015).

A common symptom of OSA is excessive daytime sleepiness, which can be described as the tendency to fall asleep during daytime (Sateia, 2014). A large American and a smaller Icelandic population-based study on subjective excessive sleepiness reported a prevalence of 8.7 and 13.1%, respectively (Bixler et al., 2005; Thorarinsdottir et al., 2019); the latter measured by a score of 11 or higher on the Epworth Sleepiness Scale (ESS). A Norwegian study based on ESS reports a 17.7% prevalence of excessive sleepiness (Pallesen et al., 2007). One of our former studies has shown excessive sleepiness in 46.5% of patients with mild OSA and 58.0% of patients with severe OSA (Bjorvatn et al., 2015).

Fatigue is described as “an imbalance between individuals’ energy potential and performance of daily activities” (Lerdal et al., 2005). In this state, individuals feel exhausted and unable to perform activities they normally expect of themselves. Thus, fatigue differs from sleepiness, as fatigue is defined by a lack of energy rather than a tendency to fall asleep. Fatigue and sleepiness are expressions that are often confused in non-scientific settings. Population-based studies on fatigue report a prevalence from 18.3 to 23.1% (Pawlikowska et al., 1994; Lerdal et al., 2005; Galland-Decker et al., 2019). It is common to assume that fatigue is associated with OSA. However, studies on patients with OSA have suggested that the fatigue is driven by depressive symptoms (Bardwell et al., 2003) or related to other underlying systemic diseases (Kaminska et al., 2012) and that it is not ameliorated by treatment of OSA with continuous positive airway pressure (CPAP; Economou et al., 2018).

It is generally accepted that there is a high prevalence of depressive symptoms in patients with OSA (Harris et al., 2009; Jackson et al., 2019). However, a study from Australia, including 109 patients with OSA, did not find an association between depression and OSA severity (Jackson et al., 2019). Furthermore, a study with 3,770 patients from our group showed that increased severity of OSA was associated with less depression and anxiety (Bjorvatn et al., 2018). The prevalence of depression in the general population has been found to be 5.2 and 5.9% in large studies from the United States and China, respectively (Ohayon, 2007; Zhou et al., 2014). Because depression is often coexistent with anxiety, we chose to include an assessment of anxiety in the present study. A Norwegian population-based study found the 12-month prevalence of generalized anxiety disorder to be 1.9% (Kringlen et al., 2001). A European literature study found the 12-month prevalence to be 0.2–4.3% (median 2.0%; Wittchen et al., 2011). A common tool for assessing depression and anxiety in a hospital setting is the Hospital Anxiety and Depression Scale (HADS; Zigmond and Snaith, 1983). A German population-based study found a prevalence of 21.0% with HADS anxiety (HADS A) score ≥8 and 23.7% with HADS depression (HADS D) score ≥8 (Hinz and Brahler, 2011).

It is essential to identify symptoms associated with OSA, as symptoms positively associated with OSA severity will increase the pretest probability of OSA and thus make it easier to prioritize patients for further testing with respiratory polygraphy or polysomnography. It may also pinpoint interesting areas for further research, as symptoms associated with OSA severity may be expected to respond to OSA treatment.

Thus, the present study aimed to investigate possible associations between fatigue and OSA and between sleepiness and OSA and whether any such associations would be related to OSA severity. We hypothesized that the prevalence of both pathological fatigue and excessive sleepiness would increase with OSA severity. As fatigue in OSA has been suggested to be driven by depressive symptoms (Bardwell et al., 2003), and depression and anxiety are often associated with each other, we also investigated whether depression and anxiety affect fatigue in patients with OSA.

## Materials and Methods

### Participant Recruitment

The study was conducted on patients referred with suspicion of OSA to the Centre for Sleep Disorders at Haukeland University Hospital in Bergen, Norway, from 2011 through 2017. All referred patients were asked to sign a consent form for research. All consenting patients (*n* = 5,631) with suspected sleep apnea were recruited. Exclusion criteria were insufficient diagnostic data from polygraphy (*n* = 167), leaving 5,464 patients for further analyses.

### Investigations

All patients underwent standard polygraphy with a type 3 portable monitor (Embletta^™^ or NOX T3, Resmed Norway AS). Most patients were recorded while sleeping at home, while a few slept in a hospital hotel. The apnea–hypopnea index/respiratory event index (AHI/REI) was autoscored by computer software but inspected and corrected manually by the sleep physician. Scoring was done in accordance with the American Academy of Sleep Medicine (AASM) Manual for the Scoring of Sleep and Associated Events version 2.4, with a 4% oxygen desaturation set as the limit for scoring hypopneas. OSA was defined as follows: Patients with AHI/REI <5 were defined as not having OSA, AHI/REI 5–14.9 as mild OSA, 15–29.9 as moderate OSA, and ≥ 30 as severe OSA (Epstein et al., 2009). Moderate-to-severe OSA (AHI/REI ≥15) was used as cutoff in some of the analyses, as the primary need for treatment is among patients with AHI ≥15 (American Academy of Sleep Medicine, 1999). Prior to polygraphy recording, all patients completed a questionnaire including Epworth Sleepiness Scale (ESS), Fatigue Severity Scale (FSS), and the Hospital Anxiety and Depression scale (HADS). Patients who did not respond to all questions in a questionnaire were excluded from all analyses directly related to that scale.

To assess sleepiness, we used the ESS (Johns, 1991). The ESS is a commonly used tool for assessing sleepiness, with eight described situations where the patient has to score the likeliness of dozing off in given situations, from 0 (no chance of dozing off) to 3 (high chance of dozing off). Sleepiness was defined using the sum of the scores for the eight different situations. A total score of 11 or higher was considered excessive sleepiness.

The FSS was used to assess fatigue. Patients were asked to assess nine different statements concerning fatigue and level them on a scale from 1 to 7, where 1 represents complete disagreement, and 7 is complete agreement with the statement (Krupp et al., 1989). The mean score from the nine statements was then calculated. A higher score indicated a higher level of fatigue. Different cutoff values for pathological fatigue are reported in the literature, with 4 being the most common (Herlofson and Larsen, 2002; Valko et al., 2008). A cutoff value of 5 was recommended in a large Norwegian study from 2005 to avoid over-diagnosing (Lerdal et al., 2005), and this was, therefore, applied in our study. However, additional analyses using a cutoff value of 4 were performed to reveal any discrepancies when applying different cutoff values.

Anxiety and depression were assessed with the Hospital Anxiety and Depression Scale (HADS; Zigmond and Snaith, 1983). HADS is a questionnaire based on the patient’s feelings during the last week. There are seven questions concerning anxiety and seven questions concerning depression, each rated on a scale from 0 to 3, with 3 being the worst. A sum score of 8 or higher on each subscale is indicative of the disorder but not diagnostic (Bjelland et al., 2002). Cutoff values of 8 were used in our analyses to separate patients with or without possible anxiety or depression, respectively.

During the consultation, weight and height were measured in order to calculate the patients’ body mass index (BMI), and BMI ≥ 30 was used as a cutoff for obesity.

### Statistics

The data were analyzed with IBM SPSS Statistics version 26 (SPSS Inc., 2019, Armonk, NY, United States). Differences in patient characteristics according to OSA severity were explored using Pearson’s chi-square tests. Statistical analyses were performed using logistic regression, with moderate-to-severe OSA as the dependent variable and sex, age, obesity, excessive sleepiness, pathological fatigue, anxiety, and depression as predictors. Furthermore, we conducted both crude and adjusted (sex, obesity, age group, excessive sleepiness, pathological fatigue, anxiety, and depression) binary logistic regressions with different OSA severity levels (mild OSA as reference) as predictors, and with excessive sleepiness, pathological fatigue, depression, and anxiety as dependent variables. We also conducted multiple linear regressions with AHI as a continuous dependent variable. The level of significance was set to 0.05. Where median is used, the data were not normally distributed, but mean is presented in the tables to give additional information.

## Results

The median age was 50 (range 16–89) years. In total, 3,801 (69.6%) were men, 2,196 (41.1%) were obese, 3,993 (75.4%) were married or cohabiting, and 1,305 were living alone.

Moderate-to-severe OSA (AHI/REI ≥15) was found in 1,833 (33.5%) of the patients. Excessive sleepiness (ESS ≥ 11) was present in 2,232 (42.7%) patients, whereas 1,852 (38.6%) of the patients were considered pathologically fatigued (FSS ≥5). Further, 985 (19.0%) patients had symptoms of depression (HADS D score ≥ 8), and 1,469 (28.4%) had symptoms of anxiety (HADS A score ≥ 8; Table 1).

**Table 1:** Background characteristics of patients referred with suspected obstructive sleep apnea (OSA) to the centre for sleep disorders at Haukeland University Hospital in Bergen, Norway (*n* = 5,464).

		Missing, *n* (%)
Male patients, *n* (%)	3,801 (69.6)	0
Age, years, median/mean (range)	50.0/49.2 (16–89)	0
Married/cohabiting patients, *n* (%)	3,993 (75.4)	166 (3.0)
Body mass index (BMI), median/mean (range)	28.9/29.7 (14.4–63.1)	158 (2.9)
Obesity (BMI ≥30), *n* (%)	2,196 (41.1)	
Smokers, *n* (%)	1,108 (21.6)	342 (6.3)
AHI/REI median/mean (range)	8.8/15.2 (0–135.2)	0
Moderate-to-severe OSA (AHI/REI ≥15), *n* (%)	1,833 (33.5)	0
Not OSA (AHI/REI <5) *n* (%)	1,845 (33.8)	0
Mild OSA (AHI/REI 5–14.99), *n* (%)	1,786 (32.7)	0
Moderate OSA (AHI/REI 15–29.99), *n* (%)	991 (18.1)	0
Severe OSA (AHI/REI ≥30), *n* (%)	842 (15.4)	0
Epworth Sleepiness Scale (ESS), median/mean (range)	10.0/9.7 (0–24.0)	235 (4.3)
Excessive sleepiness (ESS ≥11), *n* (%)	2,232 (42.7)	
Fatigue Severity Scale (FSS), median/mean (range)	4.3/4.2 (1.0–7.0)	669 (12.2)
Pathological fatigue (FSS ≥5), *n* (%)	1,852 (38.6)	
Possible depression (HADS D score ≥ 8), *n* (%)	985 (19.0)	279 (5.1)
Possible anxiety (HADS A score ≥ 8), *n* (%)	1,469 (28.4)	283 (5.2)

*AHI/REI, Apnea–hypopnea index/respiratory event index; OSA, obstructive sleep apnea; HADS, Hospital Anxiety and Depression Scale*.

While males constituted 68.4% of patients with mild OSA, this increased to 82.9% in those with severe OSA. Similarly, the percentage of obese patients increased from 42.5% (mild OSA) to 65.7% (severe OSA). Among patients aged 50–69 years, the proportion increased from 47.4% (mild OSA) to 55.8% (severe OSA), and among patients 70 years and older, the increase was from 6.8% (mild OSA) to 10.2% (severe OSA). Among patients younger than 50 years, we found an inverse association between age and OSA severity, with a higher percentage of patients with mild OSA than with severe OSA. Excessive sleepiness also increased significantly with OSA severity, from 41.2% in mild OSA to 49.7% in severe OSA. However, the proportion of patients with pathological fatigue, depression, and anxiety did not increase depending on OSA severity. Of patients with mild OSA, 38.5% had pathological fatigue; the same prevalence was seen in patients with severe OSA. The prevalence of depression and anxiety was reduced from 18.9 and 29.1% in mild OSA, respectively, to 15.0 and 19.8% in severe OSA (Table 2).

**Table 2:** Associations between different severity levels of obstructive sleep apnea (OSA) and sex, body mass index (BMI), age, excessive sleepiness, pathological fatigue, possible depression, and possible anxiety, respectively, in patients referred with suspected sleep apnea to the Centre for Sleep Disorders at Haukeland University Hospital in Bergen, Norway (*n* = 5,464).

	No OSA % (*n*)	Mild OSA % (*n*)	Moderate OSA % (*n*)	Severe OSA % (*n*)	Chi-square (df)^1^
Sex (male)	60.3 (1112)	68.4 (1221)	77.7 (770)	82.9 (698)	*178.1 (3)* ^*^
Obesity (BMI ≥30)	27.7 (491)	42.5 (738)	43.6 (421)	65.7 (546)	*342.9 (3)* ^*^
Age <30 years 30–49 years 50–69 years ≥70 years	19.7 (363) 50.1 (925) 28.1 (519) 2.1 (38)	5.9 (105) 39.9 (713) 47.4 (846) 6.8 (122)	2.1 (21) 30.2 (299) 58.7 (582) 9.0 (89)	2.7 (23) 31.2 (263) 55.8 (470) 10.2 (86)	*686.4 (9)* ^*^
Excessive sleepiness	40.6 (720)	41.2 (705)	43.4 (410)	49.7 (397)	*20.9 (3)* ^*^
Pathological fatigue	40.4 (658)	38.5 (601)	35.6 (313)	38.5 (280)	5.5 (3)^**^
Possible depression	20.8 (363)	18.9 (319)	19.3 (183)	15.0 (120)	*12.0 (3)* ^***^
Possible anxiety	32.7 (571)	29.1 (493)	26.1 (247)	19.8 (158)	*47.6 (3)* ^*^

1 *Pearson chi-square test. Significant values shown in italics*.

* *p* *< 0.001;*

** *p* *= 0.137;*

*** *p = 0.007*.

In logistic regressions, male sex, older age, obesity, and excessive sleepiness were all positively associated with moderate-to-severe OSA, both in crude and adjusted analyses. However, there was no significant association between moderate-to-severe OSA and pathological fatigue in neither crude nor adjusted analyses. Anxiety was negatively associated with moderate-to-severe OSA in crude and adjusted analyses, whereas depression was only negatively associated with moderate-to-severe OSA in the crude analysis (Table 3).

**Table 3:** Logistic regression analyses with moderate-to-severe obstructive sleep apnea [OSA with apnea–hypopnea index/respiratory event index (AHI/REI) ≥ 15] as dependent variable among 5,464 patients referred to Haukeland University Hospital in Norway with suspected OSA.

Category (*n*)	Moderate-to-severe OSA	
	Crude OR (95% CI)	Adjusted OR (95% CI)^1^
*Sex*		
Female (1,663)	ref.	
Male (3,801)	*2.24 (1.96–2.56)*	*2.71 (2.30–3.20)*
*Age*		
<30 years (512)	ref.	
30–49 years (2,200) 50–69 years (2,417) ≥70 years (335)	*3.65 (2.64–5.04)* *8.20 (5.96–11.28)* *11.63 (7.9–16.95)*	*3.06 (2.14–4.37)* *8.32 (5.85–11.84)* *13.95 (8.99–21.65)*
*Obesity BMI ≥30*		
No (3,110)	ref.	
Yes (2,196)	*2.16 (1.93–2.43)*	*2.42 (2.11–2.79)*
*Excessive sleepiness ESS ≥11*		
No (3,123)	ref.	
Yes (2,275)	*1.25 (1.11–1.40)*	*1.30 (1.13–1.49)*
*Pathological fatigue FSS ≥5*		
No (3,303)	ref.	
Yes (1,993)	0.90 (0.79–1.02)	0.92 (0.79–1.08)
*Possible depression HADS D score ≥ 8*		
No (4,200)	ref.	
Yes (985)	*0.85 (0.73–0.99)*	0.87 (0.71–1.06)
*Possible anxiety HADS A score ≥ 8*		
No (3,712)	ref.	
Yes (1,469)	*0.68 (0.59–0.77)*	*0.80 (0.67–0.95)*

*OR, Odds ratio with significant values shown in italics; CI, confidence intervals; BMI, Body Mass Index; ESS, Epworth Sleepiness Scale; FSS, Fatigue Severity Scale, HADS, Hospital Anxiety and Depression Scale*.

*^1^In the adjusted regression analyses, all parameters were entered together in the same model*.

Excessive sleepiness, pathological fatigue, depression, and anxiety were dependent variables in binary logistic regressions with different OSA levels as predictors (Table 4). Excessive sleepiness was significantly higher for patients with severe OSA as compared to mild OSA, both in crude and adjusted analyses. There were no significant associations between OSA severity (mild, moderate, or severe) and pathological fatigue. In contrast, the prevalence of depression and anxiety was significantly lower for patients with severe OSA as compared to mild OSA in both crude and adjusted analyses. Changing fatigue cutoff from FSS >5 to FSS >4 did not affect the results significantly, with one exception: With FSS > 4, there was no significant reduction of depressive symptoms after the data were adjusted for the other factors.

**Table 4:** Logistic regression with pathological fatigue, excessive sleepiness, possible depression, and possible anxiety as the dependent variables and OSA severity as covariate, among 3,619 patients referred to Haukeland University Hospital in Norway.

	Excessive sleepiness		Pathological fatigue		Possible depression		Possible anxiety	
	Crude OR (95% CI)	Adjusted OR^1^ (95% CI)	Crude OR (95% CI)	Adjusted OR^2^ (95% CI)	Crude OR (95% CI)	Adjusted OR^3^ (95% CI)	Crude OR (95% CI)	Adjusted OR^4^ (95% CI)
Mild OSA	Ref.	Ref.	Ref.	Ref.	Ref.	Ref.	Ref.	Ref.
Moderate OSA	1.09 (0.93–1.28)	1.13 (0.94–1.36)	0.88 (0.74–1.05)	0.88 (0.72–1.07)	1.04 (0.85–1.27)	1.13 (0.87–1.46)	0.85 (0.71–1.01)	0.88 (0.67–1.10)
Severe OSA	*1.41 (1.19–1.67)*	*1.36 (1.11–1.65)*	1.00 (0.83–1.20)	1.08 (0.87–1.34)	*0.75 (0.60–0.95)*	*0.70 (0.52–0.95)*	*0.59 (0.48–0.73)*	*0.68 (0.52–0.88)*

*Referred patients without OSA were excluded from the analyses shown in this table. OSA, Obstructive Sleep Apnea; OR, Odds ratio with significant values shown in italics; CI, Confidence interval*.

1 *Adjusted for age group, sex, obesity, pathological fatigue, possible depression, and possible anxiety*.

2 *Adjusted for age group, sex, obesity, excessive sleepiness, possible depression, and possible anxiety*.

3 *Adjusted for age group, sex, obesity, excessive sleepiness, pathological fatigue, and possible anxiety*.

4 *Adjusted for age group, sex, obesity, excessive sleepiness, pathological fatigue, and possible depression*.

Furthermore, we conducted multiple linear regressions with AHI (continuous parameter) as the dependent variable. These confirmed the results from binary logistic regressions, showing no significant relation between pathological fatigue and AHI (*B* = 0.76, *SE* = 0.67, *β* = 0.02, *p* = 0.254), a significant increase of excessive sleepiness with increasing AHI (*B* = 3.33, *SE* = 0.62, *β* = 0.09, *p* < 0.001) and a significant decrease of anxiety (*B* = −3.52, *SE* = 0.71, *β* = −0.09, *p* < 0.001) and depression (*B* = −1.64, *SE* = 0.81, *β* = −0.04, *p* < 0.001) with increasing AHI.

## Discussion

To the best of our knowledge, our study is the largest study on the association between fatigue and OSA, with more than 5,400 participants. Our initial hypothesis was that the prevalence of pathological fatigue and excessive sleepiness would increase with OSA severity. This was not confirmed for fatigue, as the prevalence of pathological fatigue remained the same irrespective of OSA severity.

Our data support the generally accepted notion that males and obese individuals have a higher risk of OSA and that these characteristics increase in prevalence with increasing severity of OSA. We found that male sex, older age, and obesity were the main predictors for OSA, all with odds ratios above 2.

There was a much higher prevalence of excessive sleepiness in our sample compared to the general population. Norwegian doctors are advised to refer patients with excessive sleepiness combined with snoring for OSA assessment. Excessive sleepiness has been shown to be associated with increasing OSA severity (Bjorvatn et al., 2015) and is generally considered to be a symptom of OSA (Veasey and Rosen, 2019). Our study confirms a high prevalence of excessive sleepiness among patients with OSA, and specifically a higher prevalence of excessive sleepiness in patients with severe OSA compared to patients with mild OSA, with an 8.5% difference in prevalence among the two. Still, it is worth noting that more than half of the patients with severe OSA did not have excessive sleepiness in accordance with the ESS.

Fatigue has been found to be associated with obesity, insomnia, and depression, but not with older age in the general population (Kim et al., 2017; Galland-Decker et al., 2019). Increased fatigue is seen in several patient groups, such as cancer patients (Stone et al., 2000; Berger et al., 2015), multiple sclerosis (Rooney et al., 2019), and Parkinson’s disease (Stocchi et al., 2014). Previous studies have suggested an association between fatigue and OSA (Hossain et al., 2005), but studies have also suggested that fatigue is not related to OSA severity (Kim et al., 2017). Studies have shown that the level of fatigue in OSA is driven by depressive symptoms rather than by OSA severity level, though OSA severity influenced fatigue severity (Bardwell et al., 2003, 2007). One small study suggests that fatigue in patients with OSA may be driven by low testosterone levels (Bercea et al., 2015); other studies suggest a link between increased cytokine levels and fatigue in patients with OSA (Mills et al., 2008; Zhou and Jolly, 2015).

The prevalence of pathological fatigue in our sample was 38.6% using FSS > 5, increasing to 56.0% using FSS > 4, about twice as high as the prevalence seen in general population studies. The percentage of patients with pathological fatigue was slightly lower among the patients with OSA than among the referred patients who did not meet the criteria for an OSA diagnosis and did not differ between the different severity levels of OSA, neither in crude nor in adjusted analyses. This may indicate that patients with fatigue symptoms are frequently referred to sleep clinics to assess whether their fatigue may be related to OSA.

The high prevalence of fatigue in this patient population, without any significant association between such symptoms and OSA severity, suggests that further studies are needed. A possible explanation for this high prevalence may be that referring doctors interpret them as being caused by a chronic lack of sleep. This may stimulate general practitioners to refer patients with OSA, even in the absence of other specific OSA symptoms. This view is supported by the finding of a higher prevalence of fatigue among referred patients without OSA, compared to those with an AHI/REI ≥5. Population-based studies focusing on these possible associations are warranted, as well as studies on how OSA treatment affects these symptoms. Our findings align with previous studies with smaller samples (Kim et al., 2017; Economou et al., 2018) that do not find any association between fatigue and OSA severity.

There was also a much higher prevalence of depression and anxiety in our sample compared to the general population. This may be because these conditions are associated with sleepiness and fatigue (Corfield et al., 2016), in turn raising the suspicion of OSA for the referring doctors. A literature review published in 2016 reports that anxiety severity is related to OSA severity, suggesting that treating sleep-disordered breathing may improve anxiety symptoms (Diaz and Brown, 2016). The relation between depression and OSA has been considered in former publications with conflicting results. There have been several studies finding no association between depression and OSA severity (Macey et al., 2010; Douglas et al., 2013; Bjornsdottir et al., 2016), though one study reported that depressive symptoms were positively correlated with OSA severity (Edwards et al., 2015). In our study, however, we found a negative association between mental illness and OSA severity, with a lower prevalence of depression and anxiety in patients with severe OSA as compared to patients with mild OSA. Our findings are consistent with a previous study from our group (Bjorvatn et al., 2018). Interestingly, however, in another recent study, we reported that CPAP treatment reduced symptoms of depression and anxiety in patients with OSA (Lundetrae et al., 2020). A community-based, Norwegian study from 2012 reported a negative association between OSA and psychiatric morbidity (Hrubos-Strom et al., 2012). As already discussed in one of our former studies (Bjorvatn et al., 2018), one possible reason for a lower prevalence of depression and anxiety with more severe OSA is that mental illness leading to fatigue may be perceived as OSA symptoms, leading to referral for polygraphy. If these patients do not have OSA or only mild OSA, this may lead to the false impression that severe OSA protects against mental illness. However, this hypothesis should imply that there would also be a negative association between fatigue and OSA severity, which we did not find in the present study. Another possibility is that daytime sleepiness in severe OSA leads to a light sedation, somewhat similar to the effect of benzodiazepines or sedating antidepressants that are often used to treat anxiety and depression. We have adjusted our data for daytime sleepiness and fatigue, but there may be other, similar factors that the questionnaires are not accurate enough to register. Further research on possible reasons for a negative association between depression, anxiety, and OSA severity is warranted, preferably studies from unselected samples of the general population.

### Strengths and Limitations

A major strength of the present study is the large number of patients in a prospective study. The patients were referred for obstructive sleep apnea, which was objectively measured with polygraphy. A limitation with polygraphy is that it underestimates AHI compared to polysomnography (Nerfeldt et al., 2014). The patients were further evaluated with validated questionnaires for sleepiness, fatigue, depression, and anxiety. One particular strength was that the HADS questionnaire screens for non-vegetative symptoms and has been specifically validated for OSA patients (Law et al., 2014). The response rate was high for all questionnaires. Some limitations may be pointed out: Questionnaires, though validated, are always subjective. There are objective methods for measuring sleepiness (Carskadon et al., 1986), but these are resource demanding and not fit for such large studies. There are no good, objective methods for validating fatigue, depression, and anxiety. Other validated questionnaires for these conditions exist (Fisk et al., 1994; Kroenke et al., 2010; Chilcot et al., 2016), and using more than one validated questionnaire for each condition might increase the validity of these diagnoses. However, we would argue that the combination of validated questionnaires with high response rates allow us to draw conclusions transferrable to clinical practice.

## Conclusion

Our study indicates that the prevalence of excessive sleepiness increases with increasing OSA severity, as shown in previous studies. The prevalence of pathological fatigue did not increase with increasing OSA severity, and the prevalence of anxiety or depression was lower with increasing OSA severity. However, the prevalence of fatigue, excessive sleepiness, anxiety, and depression among patients referred for suspicion of OSA was much higher than in the general population. Further studies are needed to explore possible reasons for these findings.

## Data Availability Statement

The original contributions presented in the study are included in the article/supplementary material, further inquiries can be directed to the corresponding author.

## Ethics Statement

Written informed consent was obtained from all participants included in the study. The study was in accordance with the Declaration of Helsinki and approved by The Regional Committee for Medical and Health Research Ethics of Western Norway (2014/1060 REK Vest).

## Author Contributions

KM performed the statistical analyses, interpreted the results, drafted the initial manuscript, and revised the manuscript. SL, IS, and SG interpreted the results and revised the manuscript. BB conceptualized and designed the study, interpreted the results, and revised the manuscript. All authors approved the final manuscript as submitted and agree to be accountable for all aspects of the work.

## Conflict of Interest

The authors declare that the research was conducted in the absence of any commercial or financial relationships that could be construed as a potential conflict of interest.

## Publisher’s Note

All claims expressed in this article are solely those of the authors and do not necessarily represent those of their affiliated organizations, or those of the publisher, the editors and the reviewers. Any product that may be evaluated in this article, or claim that may be made by its manufacturer, is not guaranteed or endorsed by the publisher.
